# Absence of 4-Formylaminooxyvinylglycine Production by *Pseudomonas fluorescens* WH6 Results in Resource Reallocation from Secondary Metabolite Production to Rhizocompetence

**DOI:** 10.3390/microorganisms9040717

**Published:** 2021-03-31

**Authors:** Viola A. Manning, Kristin M. Trippe

**Affiliations:** United States Department of Agriculture, Agricultural Research Service, Forage Seed and Cereal Research Unit, Corvallis, OR 97331, USA; Viola.Manning@usda.gov

**Keywords:** vinylglycine, regulation of secondary metabolites, *Pseudomonas fluorescens*, natural herbicide

## Abstract

*Pseudomonas fluorescens* WH6 produces the non-proteinogenic amino acid 4-formylaminooxyvinylglycine (FVG), a secondary metabolite with antibacterial and pre-emergent herbicidal activities. The *gvg* operon necessary for FVG production encodes eight required genes: one regulatory (*gvgR*), two of unknown functional potential (*gvgA* and *C*), three with putative biosynthetic function (*gvgF*, *H*, and *I*), and two small ORFs (*gvgB* and *G*). To gain insight into the role of GvgA and C in FVG production, we compared the transcriptome of knockout (KO) mutants of *gvgR*, *A*, and *C* to wild type (WT) to test two hypotheses: (1) GvgA and GvgC play a regulatory role in FVG production and (2) non-*gvg* cluster genes are regulated by GvgA and GvgC. Our analyses show that, collectively, 687 genes, including the *gvg* operon, are differentially expressed in all KO strains versus WT, representing >10% of the genome. Fifty-one percent of these genes were similarly regulated in all KO strains with GvgC having the greatest number of uniquely regulated genes. Additional transcriptome data suggest cluster regulation through feedback of a cluster product. We also discovered that FVG biosynthesis is regulated by L-glu, L-asp, L-gln, and L-asn and that resources are reallocated in KO strains to increase phenotypes involved in rhizocompetence including motility, biofilm formation, and denitrification. Altogether, differential transcriptome analyses of mutants suggest that regulation of the cluster is multifaceted and the absence of FVG production or its downregulation can dramatically shift the lifestyle of WH6.

## 1. Introduction

*Pseudomonas* spp. are well known for their ability to produce secondary metabolites (SM) with broad ranges of activities [1]. The *P. fluorescens* group, frequently isolated from soil and the rhizosphere, produces an array of compounds that contribute to disease suppression and plant growth promotion [2]. Some of these compounds have been adopted for use in human health and many are being explored as possible next-generation pesticides. Knowledge of how these compounds are produced and how they alter their ecosystems is fundamental if we are to harness their full potential.

**Figure 1:**
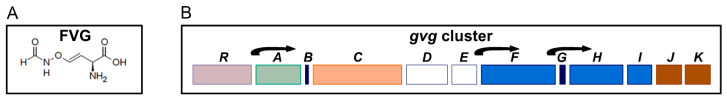
Structure of FVG and the *gvg* cluster. (**A**) Chemical structure of 4-formylaminooxyvinylglycine (FVG). (**B**) The organization of genes and promoters in the *gvg* cluster. The genes encode a putative regulator (light purple), proteins of unknown function (green, orange and dark purple), biosynthetic genes (blue), and transporters (brown). Arrows show approximate site of promoters.

*P. fluorescens* (*Pf*) strain WH6, originally isolated from the rhizosphere of wheat [3], provides a plant growth benefit when colonizing dicot roots and prevents the growth of certain species of bacteria [4,5]. *Pf* WH6 produces a non-proteinogenic amino acid analog, 4-formylaminooxyvinylglycine (FVG), with an unusual internal aminooxy bond (Figure 1A, [6]). This compound has both antibacterial and pre-emergent herbicidal activity against weedy grasses [5,7,8]. Given the dichotomy of plant growth promotion by an organism that produces an herbicidal compound, we are presented with a unique system in which to study the regulation, production, and potentially diverse ecological function of this secondary metabolite.

The biosynthetic pathway of FVG production is not known but the *gvg* cluster is required (Figure 1B, Table 1, [9,10]). In WH6, the 13-kb *gvg* cluster is composed of 12 genes, with the two central genes, *gvgD* and *E*, not necessary for FVG production. Two transporters in the LysE family, *gvgJ* and *K*, have some overlapping FVG export function, and at least one of these transporters is required for FVG export and WH6 survival. The products of the small ORFs, *gvgB* and *G*, are predicted to have ~30% lysine content and a transmembrane domain, respectively, but their function in FVG production is unclear. Of the biosynthetic genes, the putative gene product of *gvgF* is a carbamoyltransferase, *gvgH* an aminotransferase, and *gvgI* a formyltransferase. The placement of these enzymes in the biosynthetic pathway is unknown with the exception of GvgI, which is postulated to catalyze the final step in FVG synthesis with the addition of a formyl group onto aminooxyvinylglycine (AOVG). The FVG precursor produced by Δ*gvgI* mutants has similar, but not identical, herbicidal and antibacterial activities as FVG [10]. GvgA and C have conserved lipase/esterase and heme-oxygenase domains, respectively. Whether these two proteins are important for regulation or for biosynthesis is unknown. It has been suggested that they are regulatory genes as knockouts of *gvgC* in WH6 reduce transcription of *gvgH* and *I* [9] and further, knockouts of both *gvgA* and *C* in *P. chlororaphis* (*iopA* and *iopB*) results in decreased production of the secondary metabolite phenazine [11].

Regulation of transcription of the *gvg* cluster appears to be complex. GvgR is a protein in the GntR transcriptional regulator family, MocR-like subfamily. This subfamily is characterized by a helix-turn-helix DNA-binding domain and a fold type I pyridoxal phosphate (PLP)-dependent enzyme effector domain; the archetypical protein with this fold is an aspartate aminotransferase [12]. The substrate for the effector domain of GvgR is not known, yet it is worth noting that the cluster encodes an aminotransferase (*gvgH*) and that oxyvinylglycines have been shown to inhibit enzymes that require PLP as a cofactor [13]. As such, it is possible that FVG could regulate transcription of the *gvg* cluster through feedback inhibition of GvgR. Upstream of *gvgA* is a canonical GntR binding site and a full-length transcript from *gvgA* to *gvgK* is produced [10]. The presence of a full-length transcript supports the notion that GvgR regulates transcription of the entire *gvg* operon. Additionally, there are functional promoters upstream of *gvgF* and *G* (Figure 1B). Two proteins encoded outside of the *gvg* cluster, PrtI and R, also regulate FVG production. PrtI is a putative extracytoplasmic sigma factor (ECF) that negatively regulates FVG production and PrtR is thought to be an anti-sigma factor and regulates the activity of PrtI [14,15]. These genes are transcribed either as a single transcript or *prtR* independently. The environmental conditions that trigger the different transcriptional scenarios are unknown. In fact, very little is known about the environmental or physiological factors that influence FVG production.

In order to increase our knowledge of how FVG production is regulated we analyzed the transcriptomes of Δ*gvgR*, *A*, and *C* knockout (KO) strains to test the following hypotheses: (1) GvgA and C play a regulatory role in FVG production and (2) GvgA and C regulate non-*gvg* cluster genes. The data presented in the current study validate our hypotheses. We found that *gvg-* and many non-*gvg* cluster genes are similarly differentially regulated in Δ*gvgR*, *A*, and *C* strains compared to wild type (WT). The presence of a greater number of differentially expressed genes in Δ*gvgC* strains suggests a regulatory role for GvgC beyond the *gvg* cluster. We also show that feedback plays a role in cluster regulation. Examination of the functions of differentially expressed genes allowed us to discover that negatively charged amino acids and their amides negatively regulate FVG production. Furthermore, in the absence of FVG, we detected a shift from expression of genes for energy-expensive FVG production to upregulation of genes important for rhizosphere colonization. These findings imply that FVG production may be an important ecological cue for resource reallocation.

## 2. Materials and Methods

### 2.1. Culture Maintenance and Chemicals

All strains used in this work (Table S1) were maintained on Luria-Bertani (LB) Broth (Miller formulation (Becton, Dickinson and Co., Franklin Lakes, NJ, USA)) + 100 μg/mL ampicillin plates. The WH6 strain is ampicillin resistant. All chemicals were from Sigma unless otherwise noted.

### 2.2. RNA Isolation, RNAseq Library Preparation and Sequencing

For RNA isolation three mL of PMS (2.7 mM KCl, 8.7 mM NH_4_H_2_PO_4_, 16.7 mM NaH_2_PO_4_ H_2_O, 35 mM Na_2_HPO_4_, 0.04% MgSO_4_ 7H_2_0, 2 μM FeCl_3_ (stock is 1 mM in 10 mM HCl), 0.2% glucose) was inoculated with a single colony and grown overnight at 28 °C. Three replicates of 60 mL PMS in 125-mL Wheaton bottles were inoculated with 10 μL of overnight culture diluted 1:10 in PMS. Cultures were grown at 28 °C with shaking and 3 mL of each culture (OD_600_ 0.6 to 1.0) was preserved with RNAprotect (Qiagen, Redwood City, CA, USA) and stored at −70 °C until ready to process. RNA was prepared with the RNeasy Mini Kit (Qiagen), with DNA removed with the Turbo DNA-free kit (Ambion/Thermo Fisher, Waltham, MA, USA), and RNA integrity confirmed with Agilent Bioanalyzer 2100 (Agilent, Santa Clara, CA, USA) analysis on a Nano chip performed at the Center for Genome Research and Biocomputing (CGRB) Core Facility at Oregon State University. In addition, the Core Facility performed ribosomal RNA removal with the Ribo-Zero rRNA Removal Kit (Illumina, San Diego, CA, USA) and prepared libraries with the Wafergen PrepX RNA-Seq Library Kit (Takara, Mountain View, CA, USA). Libraries were multiplexed onto a single lane of an Illumina HiSeq 3000 flow cell and sequenced (100 bp, single end).

### 2.3. Differential Expression Analysis

Reads were quality trimmed with Trim Galore version 0.4.2 (www.bioinformatics.babraham.ac.uk/projects/trim_galore/, accessed on 12 June 2016), a wrapper script that runs both Cutadapt (version 1.8.1, [16]) and FastQC (version 0.11.3, [17]) post-quality trimming with minimum length = 20, quality Phred score = 20, stringency = 5, error rate = 0.1. Trimmed reads were imported into the CLC Genomics Workbench version 9.5.3. (Qiagen Digital Insights, Redwood City, CA) and mapped to genes of a manually annotated (to include *gvgB* and *G* that are not present in the current Genbank annotation) WH6 reference genome (NZ_CM001025) using default parameters (mismatch cost = 2, insertion cost = 3, deletion cost = 3, length fraction = 0.8, similarity fraction = 0.8). Principal Component Analysis based on expression levels and the underlying metadata indicated that genotype was a major source of variation between the samples and that sample prep date also contributed to the variation. The WT sample that had been prepped on a different day was removed from subsequent analyses. Differential expression of genes that differ from WT due to genotype was performed with a Generalized Linear Model assuming a Negative Binomial distribution of the read counts according to the CLC Genomics Workbench recommendations (http://resources.qiagenbioinformatics.com/manuals/advancedrnaseq/current/index.php?manual=_statistical_model.html, accessed on 30 March 2021). Dispersion estimation was performed similar to the method of Robinson et al. [18]. Genes were considered differentially expressed if the absolute value of the fold change was >2 and the False Discovery Rate (FDR) *p*-value ≤ 0.05 [19]. Venn diagrams were calculated with Venn diagram (VIB-UGent Bioinformatics and Evolutionary Genomics, Gent, BE, http://bioinformatics.psb.ugent.be/webtools/Venn/, accessed on 30 March 2021).

### 2.4. Genome Functional Annotation and Ortholog Identification

Functional annotations included protein names generated during PGAP annotation of the reference genome (NCBI, NIH), generic gene ontology (GO) slim subsets generated from merged GO term annotations based on BlastP hits (version 2.6.0+ [20], top 20 hits) and Interproscan in BLAST2GO Plug-in (version 1.9.4 [21]) of the CLC Genomics workbench, EC numbers from Interproscan, gene names from PseudoCap (version 19.1, http://www.pseudomonas.com/pseudocap [22], accessed on 30 March 2021), and manual curation. Kyoto Encyclopedia of Genes and Genomes (KEGG) annotation with KO identifiers was performed with BlastKOALA (version 2.1, 30 August 2017, [23]). Gene Set Enrichment analysis based on KEGG pathways was performed with the generally applicable gene-set/pathway analysis tool GageR (R version 3.4.2, GageR version 2.26.3, accessed 30 June 2018, [24]) using normalized, summed counts for each KO. GO term enrichment of differentially expressed genes was performed using a two-sided Fisher’s Exact test (*p*-value filter 0.1 FDR) in the Blast2GO Plug-In. COG annotations were obtained using the eggnog-mapper (http://eggnogdb.embl.de, database version 4.5.1 [25], access on 30 March 2021)

### 2.5. Quantitative Reverse-Transcriptase PCR

DNA-free RNA was prepared as above. One sample for each Δ*gvgA*, *C*, and *R* and two samples for each Δ*gvgH* and *I* strain were prepared. RNA integrity was confirmed with the Agilent TapeStation 4200 (Agilent) at the CGRB core facility. cDNA and no RT control reactions were prepared with 1 μg of RNA per reaction using the iScript Advanced cDNA Synthesis Kit (Bio-Rad, Hercules, CA, USA). Samples were diluted to 0.2 ηg μL^−1^ and stored at −80 °C. Five μL of cDNA (or no RT control) were used per droplet digital PCR reaction (ddPCR) with 0.1 μM of primers and QX200 ddPCR EvaGreen Supermix with the following conditions with all steps having a ramp 0.2 °C s^−1^: 95 °C-5 min, forty cycles 95 °C-30 s and Tm-1 min, 4 °C-5 min, 90 °C-5 min. Primer sequences and reaction Tm are shown in Table S2. Droplet preparation, PCR amplification, and droplet reading were performed at the CGRB core facility. Amplification of phosphoenol pyruvate synthase (*Pps*) cDNA was used to normalize amplification levels of target cDNAs. The no RT control was run a single time on samples with the *Pps* primers to ensure absence of gDNA contamination in RNA preps. Results were similar for the two Δ*gvgH* and *I* samples therefore only one replicate is shown.

### 2.6. Motility and Biofilm Formation Assays

For testing motility of WH6 and mutant strains 925 media [5] + 0.3% Bacto agar (Becton, Dickenson and Co) was prepared 24 h prior to plating. Strains were grown overnight in 3 mL LB medium, 28 °C with shaking. Overnight cultures were normalized to 1 OD and 2 μL of normalized culture was spotted onto the plates. Plates were incubated for 48 h at 28 °C. For testing motility on a specific N-source, M9 minimal media (48 mM Na_2_HPO_4_, 22 mM KH_2_PO_4_, 8.6 mM NaCl, 2 mM MgSO_4_, 0.1 mM CaCl_2_, 0.4% glucose) + 0.3% Noble agar (Affimetrix/Thermo Fisher) with either 3 mM L-gln or 18.7 mM NH_4_Cl were prepared 24 h prior to plating. WH6 was grown overnight in PMS media, 2 μL of overnight cultures spotted onto plates, and plates incubated for 6 days at 28 °C. Plates were scanned on an Epson Perfection V80 and area determined with ImageJ software (Rasband, W.S., ImageJ, version 1.51j8, U. S. National Institutes of Health, Bethesda, MD, USA, https://imagej.nih.gov/ij/, 1997–2018, access on 30 March 2021)

Biofilm assays were performed based on the method of O’Toole [26] with some modifications. Briefly, cultures were grown overnight from a single colony in LB. Overnight cultures were diluted 1:100 in PMS, 100 μL aliquoted into 8 wells of a 96-well culture plate and the plate incubated 3 days at 28 °C without shaking. Plates were inverted to remove cells and gently washed two times with water. Wells were stained with 125 μL 0.1% crystal violet for 10 min, rinsed three to four times, and then allowed to dry for several hours. Next, 125 μL 30% acetic acid (Spectrum, Gardena, CA, USA) in water was added to each well, incubated for 10 min, 100 μL transferred to an optically clear, flat-bottomed 96-well plate, and the plate read on an Eon (Biotek, Winooski, VT, USA) plate reader at 550 ηm with 30% acetic acid as a blank.

### 2.7. FVG-Inhibition Assay

To make culture filtrates, 10 μL of an overnight culture of WH6 grown in PMS was inoculated into 25 mL M9 + 3 mM L-aa or 18.7 mM NH_4_Cl in a 125-mL Wheaton bottle. Cultures were grown for 96 h at 28 °C with shaking. Cultures were spun down for 15 min at 4000× rpm, supernatants filtered through a 0.22 μm filter, and filtrates stored at 4 °C until used. For growth inhibition assay, *E. amylovara* was grown overnight in 3 mL LB. Culture filtrates were diluted in fresh PMS and 20 μL of filtrate and 180 μL of *E. amylovora* diluted to an OD of 0.005 were added to wells of a 96-well plate. Plates were shaken overnight at 28 °C and an OD600 reading (pathlength 0.5 cm) taken with a Synergy HT (Biotek) plate reader. Controls included a no *E. amylovora* and no filtrate.

## 3. Results

### 3.1. Absence of gvgR, A and C Result in Decreased Expression of the gvg Cluster and Large Transcriptome Changes Compared to Wild Type

#### 3.1.1. Transcriptome Differences in Δ*gvgR*, *C* and *A* Strains Compared to Wild Type

To identify genes that are regulated by GvgR, A, or C, the transcriptome of WT *Pf* WH6 was compared to that of Δ*gvgR*, *C* and *A* KO strains [10]. To accomplish this, cDNA libraries were prepared from RNA extracted from mid-log grown cultures and sequenced in a single lane of an Illumina HiSeq 3000 100 bp single end run. The reads generated per sample ranged from 23 to 37 million and were of very high quality (Table S3). Between 54–62% of trimmed and filtered reads mapped to individual genes of the WH6 reference genome and 0.03 to 0.11% of reads mapped non-specifically (Table S3).

**Figure 2:**
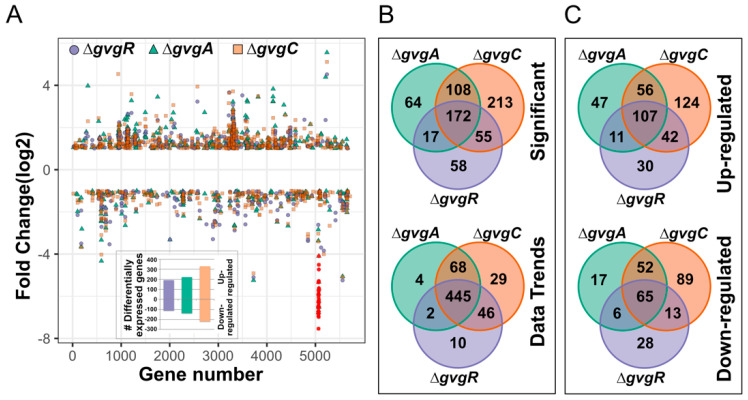
Differentially expressed genes in Δ*gvgR*, *A*, and *C* strains. (**A**) Genes significantly differentially expressed (|Fold change| > 2, FDR *p*-value < 0.05) in Δ*gvg R*, *A*, and *C* strains plotted across the WH6 genome. Red circles indicate the *gvg* cluster genes. The inset shows the numbers of up- and down-regulated genes in each knockout strain. (**B**) Overlap of differentially expressed genes for each strain. The top diagram represents the significantly different data set, while the bottom represents the manually curated data set for similar trends in the data. (**C**) Overlap of up- (top) and down-regulated (bottom) statistically significant differentially expressed genes in Δ*gvg R*, *A*, and *C* strains.

Collectively, 687 genes were significantly differentially regulated (|Fold change| > 2, FDR *p*-value < 0.05) between WT and the three mutant strains (Tables S4 and S5). In total, this number is greater than 10% of the genes in the genome. The number of differentially expressed (DE) genes between WT and the Δ*gvgC* strain was the largest at 548, with more up-regulated (329) than down-regulated (219) (Figure 2A). Fewer genes were differentially regulated in Δ*gvgR* and Δ*gvgA* strains (302 and 361, respectively) and, like Δ*gvgC*, more were up-regulated than down-regulated (up-190 and 221, down-112 and 140, respectively). Many of the significantly DE genes were similarly regulated in all of the mutants (Figure 2B) and greater that 50% of DE genes (352) were regulated similarly in at least two mutant strains (Figure 2B, top, Figure 2C). The Δ*gvgC* strain had the greatest number of specific DE genes (213), followed by Δ*gvgA* (64) and Δ*gvgR* (58). It seemed likely that the number of genes that are similarly regulated in all mutants was actually larger but the digital expression (DX) values did not meet our stringent significance criteria. To confirm this, all DE genes were manually inspected to identify low values for which differential expression is difficult to determine and to identify similar transcriptional trends (either up- or down-regulation) in all mutants compared to WT. Manual inspection revealed 275 additional loci that were similarly regulated in all mutants (Figure 2B, bottom, Table S6). After manual curation, 29 genes were DE only in the Δ*gvgC* strain and DE genes in Δ*gvgC* and one other mutant were also reduced (68 and 46, Δ*gvgA* and *R*, respectively). Very few genes were differentially expressed only in Δ*gvgA* and *R* (4 and 10, respectively). These data indicate that absence of expression of Δ*gvgA, C,* and *R* results in substantial transcriptome changes outside of the *gvg* cluster. The genes most highly upregulated in all three mutant strains included pyocin-production related genes, the nitrate assimilation transcriptional regulator *narL*, and a cluster of genes, some of which are involved in molybdenum cofactor biosynthesis (Table 2). The most highly downregulated in all mutant strains were genes in the *gvg* cluster.

#### 3.1.2. *gvg* Cluster Expression in *gvg* Gene Knockout Strains

**Figure 3:**
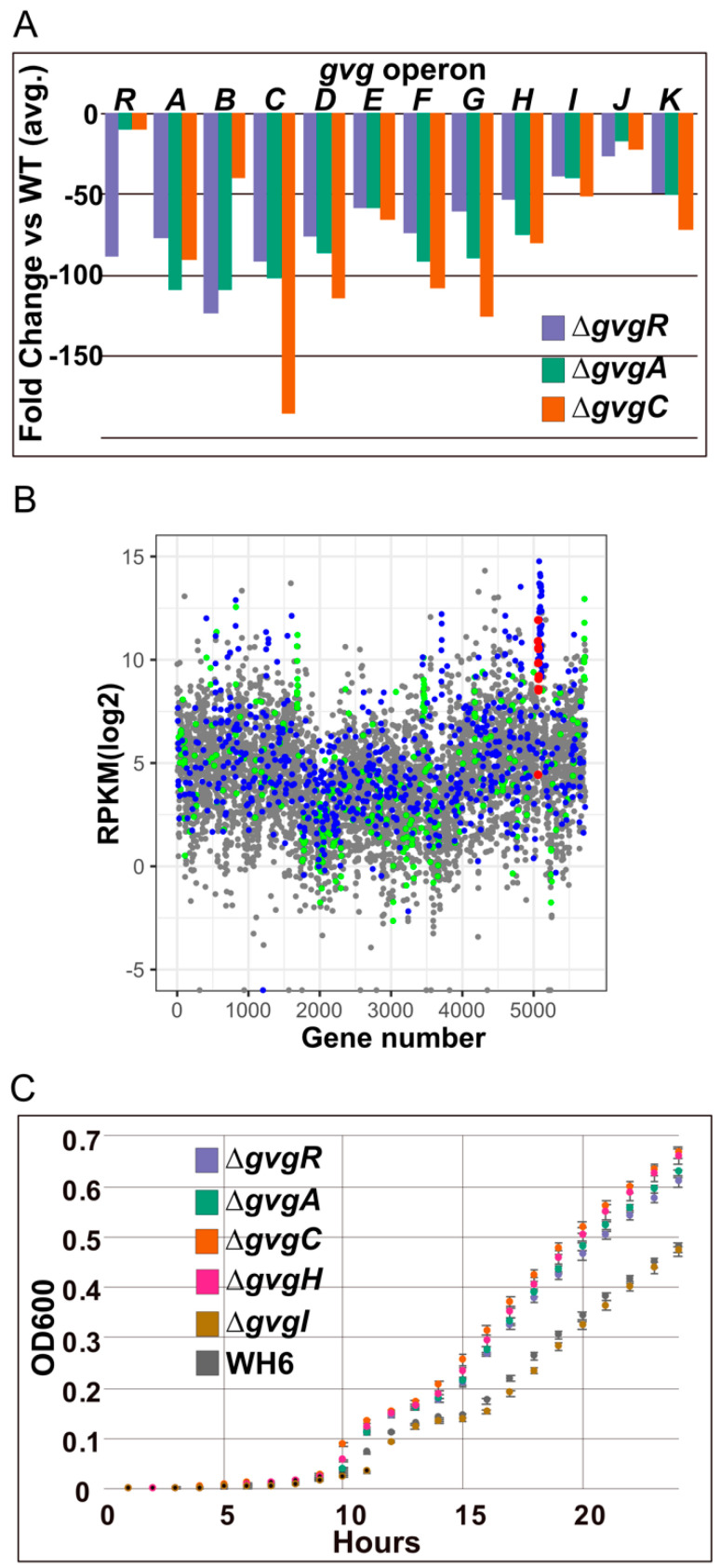
Differential expression of *gvg* cluster genes and growth of *gvg* cluster mutants. (**A**) Downregulation of all genes in the *gvg* cluster in Δ*gvg R*, *A*, and *C* strains. (**B**) Reads per kilobase of transcript, per million mapped reads (RPKM) for each gene plotted across the WH6 genome. Color coding indicates predicted gene function, including transcription and translation (blue); energy production (green), and *gvg* production (red). (**C**) Growth of *gvg* cluster mutants. Points represent a single experiment with 8 replicates. Error bars represent standard error. Two additional experiments were conducted with similar results.

Expression of all *gvg* cluster genes is massively downregulated in the Δ*gvgA*, *C* and *R* strains (Figure 3A). As expected, gene KO strains had the least expression of the corresponding gene. Expression data were confirmed by droplet digital PCR (DD) of *gvgR*, *A*, and *C* (Table 3). The average expression of genes as a percent of WT was remarkably similar for both DX and DD detection methods. For example, expression of *gvgR* in the Δ*gvgA* mutant was estimated as 9.7 and 7.5% of WT by DX and DD, respectively.

Given that DD analysis is a close proxy for DX in *gvgR*, *A*, and *C* mutants, we used DD to explore *gvg* cluster expression in additional *gvg* cluster mutants, Δ*gvgH* and *I*. The Δ*gvgH* strain does not produce an active secondary metabolite (Table 1). The Δgvg*I* strain does produce a biologically active compound, most likely AOVG, thought to be the penultimate product of FVG production. Expression of *gvg* cluster genes in *gvgH* mutants resembled that of Δ*gvgA* and *C* mutants with a massive decrease in expression compared to WT. Conversely, *gvg* cluster expression in Δ*gvgI* was similar or greater than WT. These data show that in strains with no antibacterial/herbicidal phenotype the *gvg* cluster is substantially downregulated. In addition, the data indicate that expression of *gvgR* may be directly regulated by GvgA and C*,* indirectly regulated by *gvg* cluster metabolites, or both.

GvgR is characterized by an aminotransferase effector domain. As noted above, DX and DD data from multiple *gvg* cluster KO strains, including the predicted aminotransferase Δ*gvgH* strain, suggest that a cluster metabolite modulates regulatory feedback of GvgR activity. This led us to question whether additonal MocR subfamily GntRs are also differentially regulated in knockout strains. Based on PFAM domains (Table S7) we identified 36 GntR-family transcription factors encoded by the WH6 genome. In addition to GvgR, 12 belong to the MocR subfamily (Table S8). PFWH6_RS27900 shares the highest sequence identity with GvgR (64%, Supplemental Figure S1). This locus and the two loci upstream are downregulated in Δ*gvgA*, *C*, and *R* strains (Supplemental Figure S1). PFWH6_RS27890 and RS27900 have ~ −3 and PFWH6_RS27895 > −30 fold change compared to WT. Expression level estimates of RS27890 and 95 by DD confirm decreases in Δ*gvgA*, *C*, *R* and *H* and increases in Δ*gvgI* compared to WT (Table S9). Reads from the WT and the Δ*gvgR*, *A*, and *C* strains mapped to PFWH6_RS27890-PFWH6_RS27900 indicate that the decrease in expression of PFWH6_RS27890 is due to a large decrease in an antisense transcript (Supplemental Figure S1). Several small open reading frames are predicted in the reverse orientation of PFWH6_27890 though none are the size of the transcript predicted by read mapping. To gain insight into possible operon function we used BlastP against the *P. aeruginosa* PAO1 reference database in the Pseudomonas Genome Database. The best reciprocal blast hit (BRBH) of PFWH6_RS27890 was PauB4 (PA5309), a protein involved in polyamine biosynthesis (Chou et al. 2013). The BRBH of PFWH6_RS27895 and RS27900 were a hypothetical protein (PA2031) and GntR (PA2032), respectively; unlike those in the WH6 genome, these genes are not adjacent to *pauB4* in PAO1. Attempts to knockout the PFWH6_RS27895 resulted only in merodiploids suggesting its function is necessary for cell viability. Overall, these data suggest that the two most similar MocR GntRs, GvgR and PFWH6_RS27895, are similarly regulated in Δ*gvgA*, *C*, *R* and *H*. This demonstrates that under some circumstances, i.e., low levels of *gvg* cluster expression, these GntRs and their regulons are co-ordinately regulated and polyamine metabolism and FVG production may be linked.

Analysis of the WT transcriptome indicated that levels of *gvg* gene expression were comparable to highly expressed genes necessary for transcription, translation, and energy production (Figure 3B). Abundant expression of the *gvg* cluster during FVG production is likely to require substantial consumption of cellular resources. If so, mutants with reduced *gvg* cluster expression should have faster growth rates than WT. In growth assays, Δ*gvgA*, *C*, *R*, and *H* strains had faster growth rates than the WT and Δ*gvgI* strains (Figure 3C). The mutants with increased growth rates clustered into two different groups, Δ*gvgC* with *H* and Δ*gvgR* with *A*. 

### 3.2. Functional Analysis of Transcriptome Shifts in ΔgvgR, A, and C Mutants Reveals a Role for Acidic Amino Acids and Their Amides in FVG Production

#### 3.2.1. Functional Analyses of Genes Regulated in *gvg* Cluster Mutants

**Figure 4:**
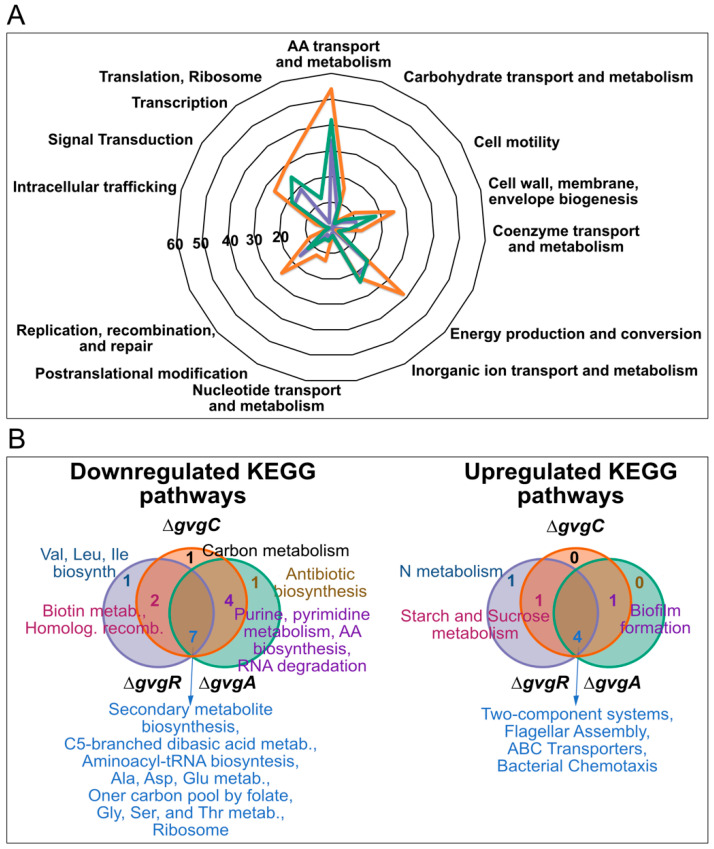
Functional annotation of differentially regulated genes in Δ*gvg R*, *A*, and *C* strains. (**A**) Radar plot of differentially regulated genes categorized into Clusters of Orthologous Groups. Labels reflect those groups which were significantly differentially regulated vs. WT and the axis indicates the numbers of genes regulated per category. Orange = Δ*gvgC,* green = Δ*gvgA*, and purple = Δ*gvgR* strains. (**B**) Venn diagrams of essential gene sets that are differentially down- (left side) or up-regulated (right side) in Δ*gvg R*, *A*, and *C* strains as determined with a gene enrichment analysis of KEGG pathway categories. The numbers and descriptions of the gene sets are shown in similar colors.

We used several methods to functionally annotate DE genes in the Δ*gvgA*, *C* and *R* strains. First, we categorized WH6 proteins into functional categories based on orthology relationships (clusters of orthologus groups (COGs), Table S10) and then determined the abundance of each category in the statistically significant datasets (Figure 4A). Across all strains, amino acid, ion, nucleotide, coenzyme, and carbohydrate transport and metabolism, cell wall/membrane/envelope biogenesis and motility, energy production, signal transduction, transcription, and translation were the most common categories assigned to DE gene products. Within these categories, the Δ*gvgC* strain had larger numbers in each category, but especially translation, transcription and energy production. A functional enrichment analysis of GO categories supports the COG categorizations of the significant dataset (Table S11).

To obtain information on biological pathways that were up- or down-regulated in the knockout strains, we performed gene set enrichment analysis based on KEGG categories (Table S12, Figure 4B). Most of the significantly regulated pathways were present in more than one knockout strain and more pathways were down- rather than up-regulated. Downregulated gene sets were associated with (1) transcription: purine and pyrimidine metabolism, and RNA degradation; (2) translation: ribosome, biosynthesis of amino acids, and tRNA biosynthesis; and (3) metabolism: carbon, biotin, amino acids, and secondary metabolites. These downregulated gene sets point toward a reduction in the need for cellular resources. Upregulated pathways were involved in biofilm formation, two-component systems, flagellar assembly and chemotaxis, transport, and nitrogen metabolism.

**Figure 5:**
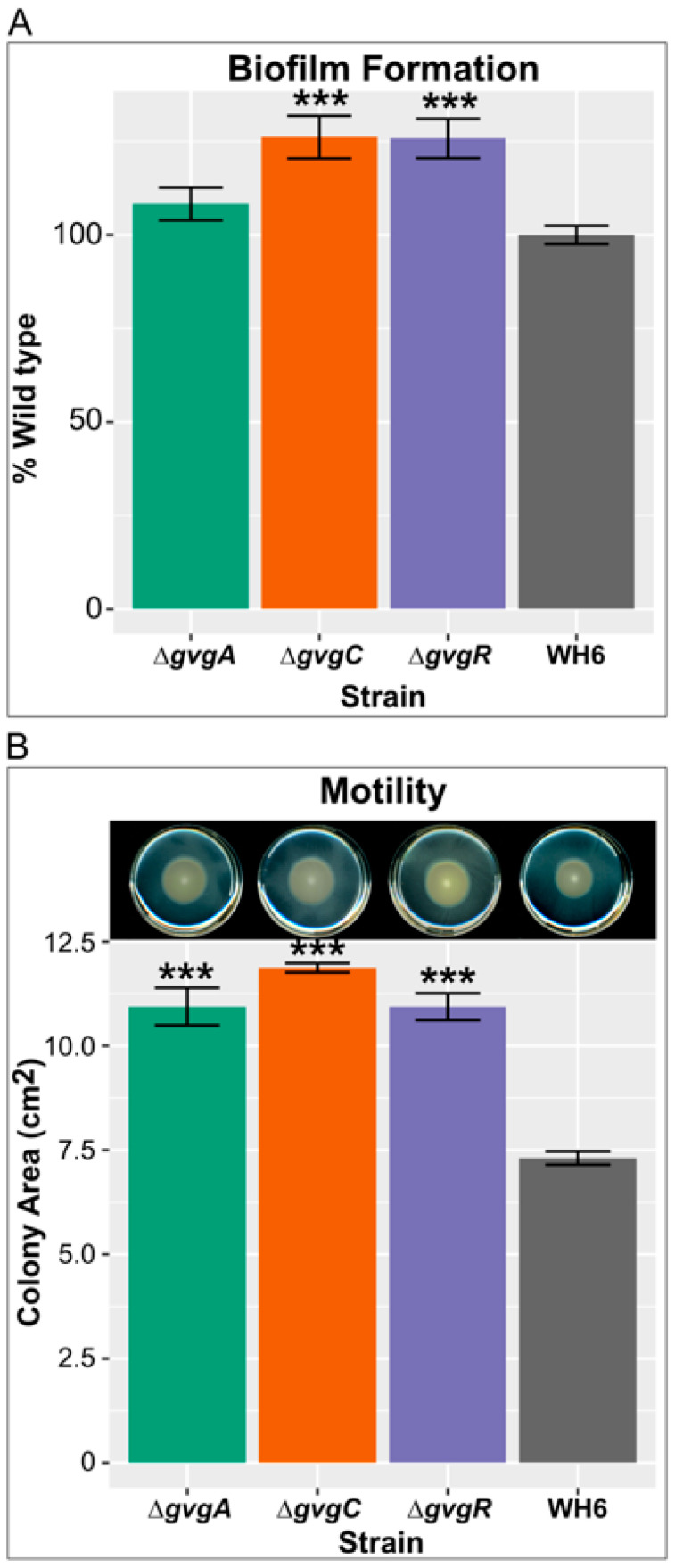
Upregulation of biofilm and flagellar biosynthesis pathways lead to increased biofilm formation and motility. (**A**) Biofilm formation as a percent of WT WH6. Bars represent the average of two independent experiments with four replicates each. (**B**) Swimming motility of Δ*gvgA*, *C*, and *R* and WT WH6. Top panel shows a representative swim plate 48 h after inoculation and the bottom panel bars represents the average area of growth after 48 h from three independent experiments with three replicates each. Error bars = standard error. *** = *p* ≤ 0.001 by Student’s t-Test.

The upregulated pathways suggested that several traits related to rhizocompetence are enhanced in the KO mutants. We therefore examined these pathways more closely and where possible determined whether their upregulation resulted in measurable changes in phenotypes. Enriched two-component systems (Table S13) related to the upregulated pathways included NarX/NarL, KinB/AlgB, AmrZ(AlgZ)/AlgR, CheA/CheY/CheB, and FleR/FleS. NarX/NarL is known to regulate denitrifying growth, arginine fermentation, and molybdenum cofactor biosynthesis [27,28,29]. The WH6 genome contains three operons for denitrification, *nar*, *nir,* and *nor*, but not the *nos* operon for conversion of nitric oxide to N [30]. All of the genes in these operons are upregulated in Δ*gvgA*, *C*, and *R* (Supplemental Figure S2). Two genes necessary for the biosynthesis of molybdenum cofactor, *moeA1* and *moeB1*, which is required for nitrate reductase activity [31], are amongst the common most highly DE genes in the KO strains (Table 2). Attempts to measure differences in nitrate and nitrite levels in the WT compared to the mutant strains yielded inconsistent results. The *arcDABC* operon (PFWH6_RS22520-22535) necessary for anaerobic arginine fermentation is also upregulated in the knockout mutants. The KinB/AlgB and AmrZ/AlgR two-component systems are known to regulate the production of alginate [32], an important component of some biofilms [33]. All genes in the *alg* operon are upregulated in each of the knockouts as are many other genes involved in alginate synthesis (Table S14, [34]). Upregulation of the *alg* operon regulator, *algD*, in Δ*gvgA*, *C*, *R*, and *H*, but not Δ*gvgI* was confirmed by DD (Table S15). Assays for biofilm detection showed more biofilm formation in *gvg* cluster mutants (Figure 5A), though only differences between Δ*gvgC* and *R* versus WT were significant. Although the CheA/CheY/CheB two-component systems that regulate chemotaxis are upregulated, of the four chemotaxis systems that WH6 contains [30], only part of the Che1 system is upregulated (PFWH6_RS20155-PFWH6_20195). FleR/FleS, as well as AmrZ regulate flagellar assembly and motility [35,36,37] and the majority of genes in the flagellar assembly pathway are upregulated in all mutants (Supplemental Figure S3). Culturing on swim media indicated a significant increase in motility in the mutant strains compared to WT (Figure 5B).

#### 3.2.2. Transportome Changes in *gvg* Cluster Mutants

**Figure 6:**
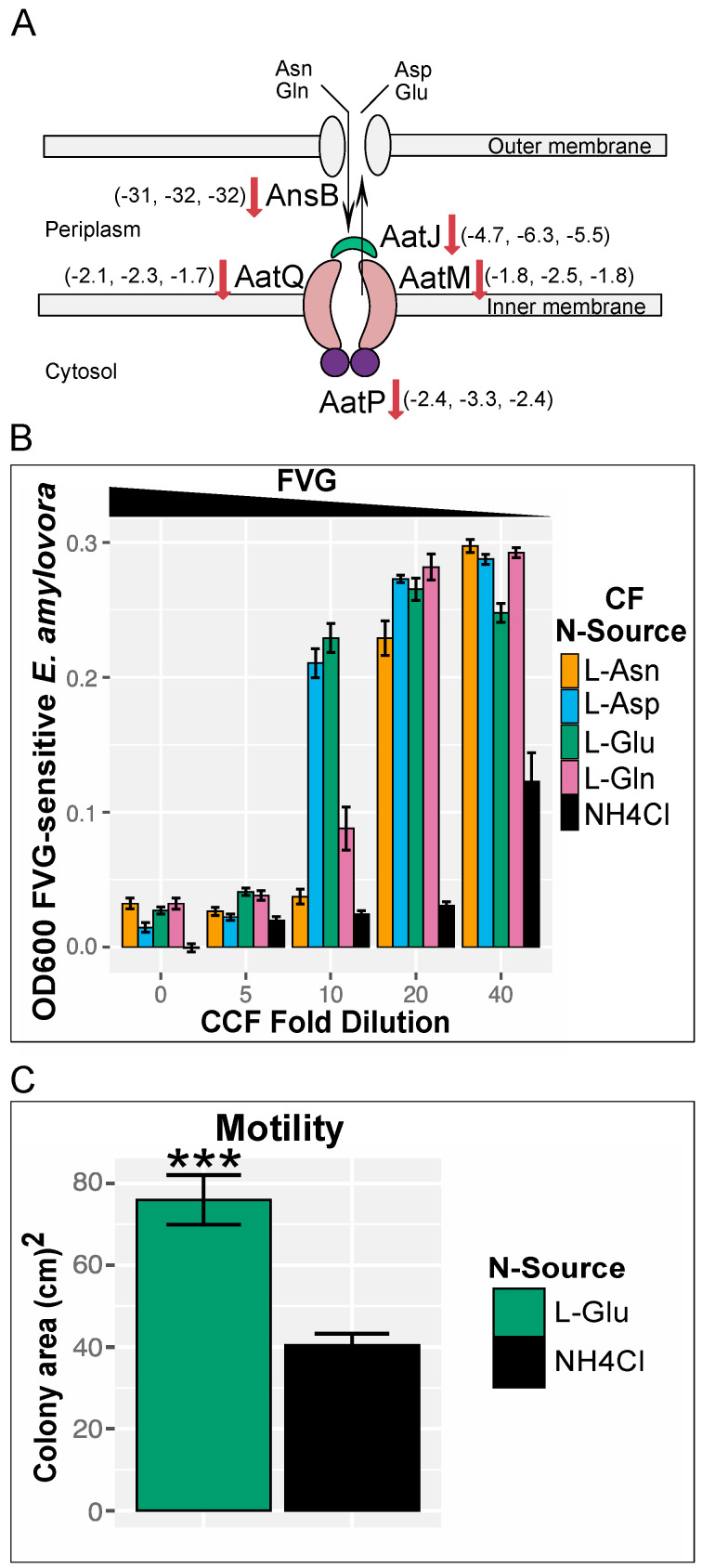
Downregulation of FVG production by acidic amino acids and their amides. (**A**) Schematic of uptake pathway of acidic amino acid and the regulation of pathway genes in *gvg* cluster mutants. The numbers in the brackets next to the gene product represent the fold difference from WT of Δ*gvgR*, *A*, and *C*, respectively. (**B**) Growth inhibition of *E. amylovora* by FVG in culture filtrates (CF) of WH6 grown in alternative N-sources. Bars represent the average of three independent experiments with six replicates each. (**C**) Swimming motility of WH6 grown in alternative N-sources after 6 days of growth. Bars represents the average colony area of three independent experiments with four to five replicates each. Error bars = standard error. *** = *p* ≤ 0.001 by Student’s t-Test.

Categorization of the DE genes into COGs as well as functional enrichment analyses of KEGG pathways and GO categories suggests that transport of biomolecules is likely altered in *gvg* cluster mutants. Fisher’s exact test (*p*-value = 1.89 × 10^−5^) of transportome loci [4] and WH6 DE loci based on KEGG orthology annotations indicated that the transportome is differentially expressed. Within the transportome, one of the most highly down-regulated genes is *ansB* [38], a glutaminase/asparaginase thought to be involved in deamidation of L-gln/L-asn during amino acid import (Figure 6A). Downregulation of *ansB* in Δ*gvgA*, *C*, *R*, and *H*, but not *I* was confirmed by DD (Table S16). The *aatJQMP* operon [39] that encodes the acidic amino acid transporter responsible for glutamate and aspartate uptake and the two-component system *aauR*/*aauS* that regulates both the *aatJQMP* operon (Singh and Rohm 2008) and *ansB* are also downregulated.

The downregulation of genes involved in the uptake of acidic amino acids and their amides in *gvg* cluster mutants suggests these amino acids may be involved in regulation of FVG production. To test this, we designed a dose-dependent growth inhibition assay utilizing an FVG-sensitive bacterial strain as the FVG sensor. It was previously shown that certain strains of *Erwinia amylovora* [5,40] that contain a particular allele of the asparagine permease gene, *ansP* [41], are sensitive to FVG. Further, we have shown that the amount of FVG present in culture filtrates (CF) induces concentration-dependent growth inhibition in FVG-sensitive *E. amylovora* [8]. In our newly developed assay, we collected CF from WH6 cultured with relevant amino acids as a sole N-source and monitored the growth of FVG-sensitive *E. amylovora* in the presence of serial dilutions of the CF. In this assay, the amount of *E. amylovora* growth is inversely proportional to the amount of FVG in the CF (Figure 6B). All undiluted and 1:5 diluted CFs severely inhibited *E. amylovora* growth. Amendment of cultures with control CF from WH6 grown with NH_4_Cl as the sole N source showed increased growth only at the highest CF dilution of 1:40. However, increased growth of *E. amylovora* occurred in cultures amended with a 1:10 dilution of CF from WH6 grown in L-asp and L-glu and at a 1:20 dilution of CF from L-asn- and L-gln-grown cultures. Therefore, the amount of FVG produced with different N-sources is NH_4_Cl > L-asn, L-gln > L-asp, L-glu. As the absence of FVG production results in increased motility (Figure 5B), growth on a particular amino acid that decreases FVG production should also increase motility. WH6 grown on swim media with L-glu as the sole N-source is more motile than when grown with NH_4_Cl (Figure 6C).

## 4. Discussion

There have been no commercial herbicides with new modes of action produced in the last few decades [42]. The herbicidal activities of SM produced by microbes are excellent candidates for new discoveries to fill this need. FVG and other oxyvinylglycines offer a new pre-emergent herbicidal alternative but production of these compounds will need to be cost-effective for them to be adopted. Due to the unusual amino-oxy bond of FVG, chemical synthesis remains elusive so that at this time only large-scale fermentation is an option. However, despite the high rate of expression of the FVG cluster in log-phase minimal media cultures, concentrations of FVG in CF remain low. Understanding the regulatory mechanisms that control FVG production can inform efforts to scale production. 

### 4.1. Regulation of the gvg Operon

In this study we confirmed our hypothesis that GvgA and C play a regulatory role in FVG production. Given that there are large numbers of similarly regulated genes in Δ*gvgA*, *C*, and *R* and the biosynthetic gene *gvgH* also has reduced *gvg* cluster expression it is difficult to disentangle the mechanism of GvgA and C regulation. We did however discover that the absence of FVG production in Δ*gvgA*, *C*, and *R* is due to a decrease in transcription of the entire cluster. The reduction of *gvg* cluster expression in Δ*gvgR* strains indicates that the GvgR transcription factor (TF) is a positive regulator of cluster transcription. The MocR-subfamily of TFs are chimeric proteins that generally operate as dimers and may act as positive or negative regulators (reviewed by [12]). In many members of the subfamily, the aspartate aminotransferase-like effector-binding region contains conserved amino acid residues required for binding of PLP. For some MocR TFs, PLP alone acts as the effector; these TFs are generally involved in vitamin B6 metabolism. Others have additional binding sites that act as sensors for up- or down-regulating the catabolic pathways in which they are involved and many of the known effectors contain amino groups. For example, to prevent toxic levels of GABA from accumulating, GabR binds both PLP and GABA to positively regulate expression of GABA-catabolic genes. In this and other examples, the effector is the target of the pathway and the TF acts as an activator. In other examples, the effector is a catabolite of the pathway and the TF acts as a repressor. GvgR has the conserved amino acid residues for PLP binding (Supplemental Figure S1) but the effector that regulates its activity is unknown. Our transcriptome analyses show that *gvg* cluster genes have low expression levels in strains that do not produce an antibacterial/herbicidal product and are highly expressed in Δ*gvgI* and WT strains. This suggests that a byproduct of FVG biosynthesis is an effector of the pathway. As GvgH and the effector-binding region of GvgR both have aminotransferase domains it is intriguing to think that GvgH activity might be responsible for effector accumulation required for cluster regulation. Involvement in production of a secondary metabolite makes GvgR unique amongst the currently characterized MocR TFs.

Another MocR TF, PFWH6_RS27900, and two upstream genes are down-regulated similarly in the Δ*gvgR*, *A*, and *C* strains. The effector binding domain shows a high degree of similarity between GvgR and RS27900. As is common for secondary metabolite gene clusters [2], the *gvg* cluster is limited to only a few *Pf* strains [43], whereas the RS27890-RS27900 gene cluster is highly conserved (data not shown). The function of the RS27890-RS27900 cluster is not known but RS27890 encodes the putative ortholog of *P. aeruginosa pauB4* (PA5309) whose gene product is involved in polyamine catabolism. PauB4 is a part of the γ-glutamylation pathway, being required for growth on spermidine [44]. However, *pauB4* in *P. aeruginosa* is in a different genomic context than in *Pf* with no adjacent hypothetical protein or GntR homologous to RS27895 and RS27900. These observations suggest different regulation and possibly function of this gene in the two species. Other MocR TFs, specifically the GabR and OapR group of regulators, have been associated with regulons involved in GABA utilization, including the γ-glutamylation pathway for polyamine degradation [45]. Co-regulation of these two clusters offers the possibility that polyamine metabolism and FVG production could be linked.

As with regulation of FVG production, we confirmed that GvgA and C regulate non-*gvg* cluster genes; however, we could not show that these gene products were direct regulators of gene expression. Rather, we showed that large numbers of genes are differentially regulated in WT WH6 versus Δ*gvgR*, *A*, and *C* strains. The number of differentially regulated genes in these mutants is similar to the number of differentially expressed genes (>10%) in a mutant of the global regulator *gacA* in *P. protogens* Pf-5 [46]. However, neither *gacA* nor the corresponding sensor kinase gene *gacS* is differentially regulated in any of the *gvg* KO mutants. A comparison of DE genes in *P. chlororaphis* 30-84Δ*gacA* vs. WT [47] and WH6 *gvg* KO strains vs. WT shows ~10% overlap in regulated genes with ~ half of these genes differentially regulated in the opposite manner (data not shown). The GacA/S system, therefore, does not seem to control the massive transcriptional consequences of FVG production.

Mutant WH6 strains that do not produce an antibacterial/herbicidal compound from the *gvg* cluster grow at faster rates than WT and many down-regulated genes in these strains are clearly involved in providing the resources necessary for their production. Their faster growth rate is concomitant with a large decrease in the transcription of the *gvg* cluster and in genes involved in transcriptional, translational, and energy-producing machinery. Production of FVG and the likely precursor AOVG from the *gvg* cluster, therefore, requires abundant cellular resources. The signal for increasing metabolic functions necessary for production of FVG is unknown, however, Δ*gvgC* strains have greater numbers of downregulated genes involved in transcription, translation and energy production than the other KO strains suggesting the possibility of GvgC’s involvement.

### 4.2. The Transcriptome of gvg Cluster Mutants Reveals Involvement of Acidic Amino Acids and Their Amides in the Regulation of FVG Production

Examination of the functions of the large number of genes differentially expressed in *gvg* cluster mutants allowed us to glean insights into FVG production. Due to the downregulation of a number of genes involved in uptake of negatively charged amino acids and their amides in Δ*gvgR*, *A*, and *C* strains, we questioned whether these amino acids regulate FVG production. If acidic amino acids are positive regulators, we would have expected that growth with any one of these amino acids as the sole N-source would lead to increased FVG production. Instead we found the opposite, that both acidic amino acids and their amides negatively regulate FVG production, with the acidic amino acids having a greater effect. This suggests that the down-regulation of the import apparatus of these amino acids in FVG¯ strains may be to limit the uptake of a negative regulator. How these amino acids might regulate FVG production and the significance of decreasing production of FVG in their presence is unknown. However, acidic amino acids and their amides may modulate FVG expression in the rhizosphere as they are exuded by roots [48,49]. Further, it has been shown that microbial products can enhance efflux of amino acids from roots [50]. Taken together with our findings that the WH6 transportome is significantly different in FVG¯ strains, a picture emerges of complex cross-talk between the plant root and WH6. 

### 4.3. Genes Involved in Rhizocompetence Are Upregulated in the Absence of FVG

In addition to gaining understanding of *gvg* cluster regulation, the impact of FVG production on the transcriptome, and the regulation of FVG production by acidic amino acids, we observed that genes necessary for rhizocompetence phenotypes were positively regulated in the absence (or decrease) of FVG. There are several well-documented competence traits common to rhizosphere-colonizing bacteria [51,52,53,54,55]. Motility in *Pf* is important for root colonization [56] and highly motile variants have a competitive advantage [57]. Chemotaxis towards particular root exudate compounds, including amino acids, by *Pf* strains has also been shown to be important for rhizosphere colonization [58,59]. Our differential expression data indicate that Δ*gvgR*, *A*, and *C* strains have increased transcription of genes necessary for motility and chemotaxis and phenotypic analysis shows these mutants are more motile. In addition, in the presence of glutamate, an amino acid shown to be abundant in some root exudates [48,49], motility of WT WH6 increases while production of FVG decreases. This suggests a tradeoff between FVG production and increased capacity for rhizosphere colonization. As FVG has herbicidal activities, reducing its production early in the root/WH6 interaction may be an important necessary first step. Biofilms have also been shown to be important in plant-microbe interactions [60] and production of biofilms on roots is characteristic of some plant growth promoting (PGP) bacteria [61,62]. The *alg* operon, which is necessary for the production of the biofilm polysaccharide alginate [32,33,63], is upregulated in Δ*gvgR*, *A*, and *C* strains. In culture, WH6 does not form extensive biofilms; however, in a standard biofilm assay of stationary cultures the Δ*gvgR*, *A*, and *C* strains produce a more measurable biofilm than WT WH6. Consistent with this ability of WH6 to form biofilms under alternative conditions (i.e., decreased FVG production), WH6 has also been shown to produce dense biofilms on aspen roots [62]. In addition to motility, chemotaxis and biofilm production, the ability to reduce nitrate and nitrite has been shown to confer an advantage in rhizosphere colonization [64,65]. As with the other rhizocompetence traits, expression from the gene clusters involved in denitrification in Δ*gvgR*, *A*, and *C* strains are increased. Bacteriocins produced by pseudomonads, including pyocins, are antimicrobial compounds that affect bacteria closely related to the producer, thus providing a competitive advantage [66,67]. It has been shown that pyocin production in *P. chlororaphis* 30–84 enhances persistence in the rhizosphere [68]. Though it is not known if WH6 produces a pyocin, genes known to be involved in pyocin production are amongst the most highly upregulated in KO strains. In combination, these data suggest that as WH6 senses specific components of root exudates, expression of the *gvg* cluster is downregulated and rhizocompetence genes are upregulated to enhance colonization of the rhizosphere.

Schinde et al. [4] showed that WH6 has PGP activity and suggested that *Pseudomonas* strain-specific differences in PGP may be due to transportomic capacity. Additionally, they postulate that the transportome can impact nutrient acquisition, root architecture, and rhizosphere colonization. Aspen seedlings grown with WH6 had increased rootlets and root biomass compared to uninoculated control plants. Increased rootlets increase the surface area of roots to allow for greater nutrient acquisition and bacterial colonization. Correlation network analysis of plant phenotypes and transportomic capabilities showed that WH6-transportomic capabilities were positively associated with rootlet formation. Bacterially produced compounds that are known to influence root morphology include polyamines [69], glutamate [70], auxin [71], and compounds that modulate auxin levels in plants [72]. Interestingly, our transcriptome data links a decrease in FVG production to the decrease in an antisense transcript that may play a role in repression of polyamine metabolism. Furthermore, WH6 has enhanced transportomic capacity for several ligands, including L-glutamate and the polyamine cadaverine. Unlike other PGP pseudomonads, WH6 does not produce auxin, though it may modulate auxin’s effects through the activity of FVG (see below).

Ethylene has a wide range of biological activities, playing a role in root growth by modulating auxin synthesis and distribution [73]. Additionally, it has been shown that bacteria with PGP activity can modulate plant ethylene levels [72]. Some PGP bacteria are known to produce 1-aminocyclopropane-1-carboxylic acid (ACC) deaminase that hydrolyses ACC, the immediate precursor of ethylene, leading to a reduction in ethylene [74] and increased root length [75]. In other cases, PGP can increase ethylene accumulation resulting in increased root hair production and greater root surface area [76]. This is reminiscent of the root phenotype seen in Aspen co-cultured with WH6 [4] and what we have seen in laboratory experiments with perennial ryegrass cultured with WH6 (data not shown). This result is actually opposite of what we would expect if FVG has similar ethylene-inhibitory activity to the FVG-related compound aminoethoxyvinylglycine (AVG) [77]. However, it has been shown that chronic treatment of roots with high concentrations of AVG leads to decreased root length [78] and therefore the impact of FVG on root phenotype could be due to length of exposure and/or concentration.

In summary, we have shown that reduction of expression of the *gvg* cluster leads to extensive reprogramming of the WH6 transcriptome away from the costly expression of the cluster towards expression of genes involved in rhizocompetence. We have also uncovered additional complexities in the regulation of the *gvg* cluster that involve more genes in the cluster than the MocR transcriptional regulator GvgR. While we were unable to determine whether GvgC and A are regulatory or biosynthetic proteins, the product of GvgC appears to have greater impacts on the transcriptome than either GvgR or A, suggesting a role in processes other than FVG production. A decrease in production of FVG with a concomitant increase in motility in the presence amino acids present in root exudates leads to speculation that WH6 can shift between FVG production and rhizocompetence depending on environment. These observations lead to many new questions. First, does WH6 promote growth in monocots as it does in dicots and is FVG involved? This seems counterintuitive given the herbicidal activity of FVG. Second, are WH6 and closely related strains present predominantly in agricultural systems due to selection of plant growth promoting bacteria? Third, does the transcriptome of WH6 differ in bulk soils versus rhizosphere soils: is there a shift away from FVG production towards rhizocompetence and are these shifts induced by root exudates? Forth, what is necessary for feedback regulation of *gvg* cluster expression? Finally, given that MocR transcription factors that positively regulate clusters are generally associated with catabolic pathways, is the *gvg* cluster actually a catabolic pathway?

## Figures and Tables

**Table 1:** *gvg* operon loci conserved domains, putative function, and mutant phenotype.

NZ_CM001025.1 (CM001025.1)	Gene	Protein Features/ Conserved Domains	Putative Function	Antibacterial/Herbicidal Phenotype of Knockout ^a^
PFWH6_RS25400 (PFWH6_5248)	*gvgR*	Aminotransferase class I and II; helix-turn-helix DNA-binding domain	GntR transcriptional regulator	−
PFWH6_RS25405 (PFWH6_5249)	*gvgA*	Isoamyl acetate hydrolase-like	Lipase/esterase	−
(PFWH6_5250)	*gvgB*	Lysine rich		−
PFWH6_RS25410 (PFWH6_5251)	*gvgC*	Heme-oxygenase 2	Redox enzyme	−
PFWH6_RS25415 (PHWH6_5252)	*gvgD*	Amidinotransferase	Amidinotransferase	+
PFWH6_RS25420 (PHWH6_5253)	*gvgE*	LysE type translocator	Amino acid exporter	+
PFWH6_RS25425 (PHWH6_5254)	*gvgF*	NodU family carbamoyltransferase	Carbamoyltransferase	−
(PHWH6_5255)	*gvgG*	Signal peptide		−
PFWH6_RS25430 (PHWH6_5256)	*gvgH*	Aminotransferase class III	Aminotransferase	−
PFWH6_RS25435 (PHWH6_5257)	*gvgI*	Formyltransferase	Formyltransferase	+
PFWH6_RS25440 (PFWH6_5258)	*gvgJ*	LysE type translocator	Amino acid exporter	+
PFWH6_RS25445 (PHWH6_5259)	*gvgK*	LysE type translocator	Amino acid exporter	+

^a^ (+) is positive and (−) negative for phenotype.

**Table 2:** Top ten similarly regulated genes in *gvg* cluster mutants compared to wild type WH6.

	Strain					
Locus Tag	Δ*gvgA*	Δ*gvgC*	Δ*gvgR*	Gene Name	Annotation	Pathway
Upregulated						
PFWH6_RS05650	5.51	3.46	3.64		pyocin terminase, ssu (pyocin cluster)	
PFWH6_RS05685	6.12	3.42	3.82		hypothetical protein	pyocin cluster
PFWH6_RS05705	5.96	3.81	6.26		hypothetical protein	pyocin cluster
PFWH6_RS10425	6.49	8.37	4.13		aldehyde dehydrogenase	
PFWH6_RS16565	5.67	6.05	5.17	*narL*	transcriptional regulator	nitrate assimilation
PFWH6_RS16575	9.31	13.15	8.42	*yhbT*	lipid carrier protein	
PFWH6_RS16580	5.44	6.72	6.8	*yhbV*	protease	
PFWH6_RS16585	7.26	7.23	6.43	*yhbU*	protease	
PFWH6_RS16590	5.26	4.86	5.65	*moeA1*	molybdenum cofactor biosynthesis protein A	molybdenum cofactor biosynthesis
PFWH6_RS16595	5.69	6.67	6.11	*moaB1*	molybdopterin biosynthesis protein B	molybdenum cofactor biosynthesis
Downregulated						
gvgB	−109.44	−40.51	−123.51	*gvgB*		FVG biosynthesis
PFWH6_RS25405	−109.25	−90.91	−77.07	*gvgA*	lipase/esterase	FVG biosynthesis
PFWH6_RS25410	−102.18	−185.79	−91.89	*gvgC*	redox enzyme	FVG biosynthesis
PFWH6_RS25425	−92.04	−107.74	−74.38	*gvgF*	Carbamoyltransferase	FVG biosynthesis
gvgG	−89.21	−126.14	−60.98	*gvgG*	secreted protein	FVG biosynthesis
PFWH6_RS25415	−87.03	−113.93	−76.31	*gvgD*	amidinotransferase	FVG biosynthesis
PFWH6_RS25430	−75.3	−80.03	−53.95	*gvgH*	aminotransferase	FVG biosynthesis
PFWH6_RS25420	−59.1	−65.66	−58.76	*gvgE*	LysE transporter	FVG biosynthesis
PFWH6_RS25445	−50.13	−71.72	−49.13	*gvgK*	LysE transporter	FVG biosynthesis
PFWH6_RS25435	−40.1	−51.65	−38.8	*gvgI*	formyltransferase	FVG biosynthesis

**Table 3:** Confirmation of *gvg* cluster gene expression vs. wild type in *gvg* cluster mutants via droplet digital PCR.

	Digital Expression (cpm ^a^) Average % WT			Droplet Digital Expression Average % WT				
Gene Detected	Δ*gvgA*	Δ*gvgC*	Δ*gvgR*	Δ*gvgA*	Δ*gvgC*	Δ*gvgR*	Δ*gvgH*	Δ*gvgI*
*gvgR*	9.7	9.5	1.1	7.5	9.5	0.4	9.2	178.6
*gvgA*	0.9	1.1	1.3	0.0	0.5	0.7	0.3	138.3
*gvgC*	1.0	0.5	1.1	0.2	0.0	0.6	0.4	130.4
*gvgH*	1.3	1.2	1.9	0.6	0.6	1.0	0.0	111.3
*gvgI*	2.5	1.9	2.6	0.4	0.6	1.0	0.8	0.0

^a^ Counts per million.

## Data Availability

High throughput sequencing reads have been deposited in the SRA database at the NCBI under Bioproject PRJNA702810.

## Supplementary Materials

The following are available online at https://www.mdpi.com/article/10.3390/microorganisms9040717/s1, Figure S1: Comparison of GvgR and MocR RS27900, Figure S2: Plotting of fold change expression of denitrification genes in Δ*gvgR*, *A*, and *C* strains compared to WT, Figure S3: Representative example of differentially expressed flagellar assembly genes mapped onto the flagellar assembly KEGG pathway, Supplementary Table S1. Bacterial strains used in this study; Table S2. Primers used in this study; Table S3. Sequencing and mapping statistics; Table S4. Locus identification, expression, GO categorization and annotation; Table S5. Statistically significant differentially expressed loci; Table S6. Manually curated differentially expressed loci; Table S7. PFAM domains in WH6 proteins; Table S8. GntR loci in WH6; Table S9. Droplet digital confirmation of differential expression of MocR operon genes; Table S10. COG categorization of WH6 loci; Table S11. Enriched GO categoris in the differentially expressed significant dataset; Table S12. KEGG ortholog identifiers for WH6 loci; Table S13. Differentially regulated two-component systems; Table S14. Differential expression of loci involved in alginate production; Table S15. Droplet digital confirmation of differential expression of *algD*; Table S16. Droplet digital confirmation of differential expression of *ansB*.
