# Supplementary material for: Absence of 4-Formylaminooxyvinylglycine Production by Pseudomonas fluorescens WH6 Results in Resource Reallocation from Secondary Metabolite Production to Rhizocompetence
Source: Microorganisms. 2021 Mar 31;9(4):717. doi: 10.3390/microorganisms9040717 (PMC8067088; doi:10.3390/microorganisms9040717)
Supplement: Supplementary file 1 [file microorganisms-09-00717-s001.zip › Supplemental/Supplemental1.pdf]

A. Amino acid comparison of MocR TFs in WH6

Amino Acid Percent Identity (upper triangle); Amino Acid Differences (lower triangle)

WH6 locus		1	2	3	4	5	6	7	8	9	10	11	12	13
GvgR	1		63.92	35.80	36.81	21.83	20.75	24.51	21.34	20.15	17.51	19.31	18.43	16.18
RS27900	2	175		39.29	39.50	22.27	21.78	25.60	23.82	21.90	17.01	17.50	18.22	16.12
RS12370	3	312	289		39.92	23.65	22.55	21.04	19.66	19.61	16.60	19.81	19.96	15.29
RS21500	4	309	291	289		22.42	23.32	20.75	19.77	20.81	17.70	20.31	18.15	15.77
RS08645	5	394	391	381	391		32.51	20.19	19.52	16.38	16.76	17.97	19.61	15.66
RS12515	6	401	395	388	388	328		18.52	18.44	17.56	18.80	19.06	19.13	15.61
RS15445	7	388	375	394	401	411	418		35.70	29.07	17.46	17.59	15.99	14.66
RS28335	8	424	403	421	426	433	438	335		34.98	16.76	18.68	15.78	15.17
RS19630	9	420	403	410	411	439	432	366	342		17.01	14.53	13.99	14.26
RS21270	10	438	439	437	437	432	419	435	452	444		35.61	19.96	19.57
RS21540	11	422	429	413	416	420	412	431	444	459	311		20.93	18.77
RS13985	12	425	422	409	424	410	410	436	459	461	405	389		13.18
RS15350	13	435	432	432	438	431	438	454	464	457	415	411	448	

B. Protein alignment of GvgR and RS27900

```
GvgR     MTLYLNLAEF ISSRIEQGLY GPGERLPSVR TLSHEHGVSL TTVQQAYRVL ECSGLAIPRP KSGYFVPSDR RVAPLPQMGR PILRPVDISQ 90
RS27900  MTLYVNLAEL LGTRIEQGFY RPGDRLPSVR ALSVEHGVSL STVQQAYRLL EDNGLAMPKP KSGYFVPVGR ELPALPEVGR PAQRPVEISQ 90

GvgR     WDMVQELMRF DQSADIVQLG CGMPDISAPT LKPLLTAMAR ISRRSDSTSL QYSHIQGVLA LREQIARLSI DSGVRLTPAD LIITSGCQEA 180
RS27900  WDQVLELIRA VPRKGVIQLG RGMPDVLSPT LKPLLRSLAR VSRRQDLPGL YYDNILGCME LREQIARLSL DSGCQLSAED IVITTGCHEA 180

GvgR     LSTAIRAICS PGDIVAVASP SYHGMMQILK AADLKALEIP SDPVTGINLE ALEMALEQWP IKVIQLTANC NNPMGYIMPD ERKLKLIRMA 270
RS27900  LSASIHAICE PGDIVAVDSP SFHGAMQTLK GLGMKALEIP TDPITGISLE ALELALEQWP IKVIQLTPNC NNPLGYIMPE ARKRALLTLA 270

GvgR     QRFDVPIIED D YGDLSHRY PRPRSLKSFD EDNRVVLCSS LSKTVAPGLR VGWIAPGRYL PQVLHMKYIG SGATVTQPQL AAAEFIAKGN 360
RS27900  QRFDVAIIED DVYGELAYSY PRPRTIKSFD EDGRVLLCSS FSKTLAPGLR IGWVAPGRYL DRVLHMKYIS TGSTATQPQI AIAEFLKNGH 360

GvgR     YLLHLRRMRK QYKRNGDLMS EWVNRYFPAG TRVSQPQGGF TLWVELPGEF DTYALNRLLE AQDVQVANGN IFSAAGKYRN CIRLNFANPP 450
RS27900  FEPHLRRMRT QYQRNRDLML DWVSRYFPAG TRASRPQGSF MLWIELPDGF DTLKLNRALI EQGVQVAVGS IFSASGKYRN CLRMNYSAKP 450

GvgR     TRKVEMAVQK VGQTVGQLMQ MESDNTLTAQ QRQP       485
RS27900  TPQIEEAVRK VGAAASKILA ESVD*----- ----       475
```

C. Mapping of reads from WT and Δ*gvgR* strains to the RS27900 regulon

RS27900 regulon

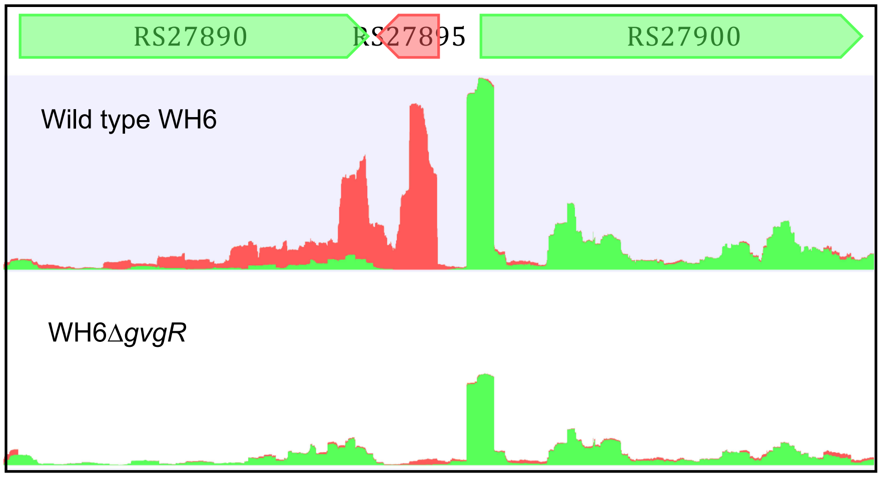

Supplemental Figure 1. Comparison of GvgR and the MocR RS27900. (A) Amino acid comparison between MocR's in WH6. (B) Protein alignment of the two most similar MocR's GvgR and RS27900. Black residues are identical. Amino acids circled in red are conserved PLP-binding residues. (C) Mapping of sequencing reads from wild type WH6 and ΔgvgR strains to the RS27900 regulon. Arrowheads on the genes point to direction of transcription. Green = reads map to sense strand and red = reads map to anti-sense strand. From CLC Genomics Workbench.
