# Supplementary material for: Absence of 4-Formylaminooxyvinylglycine Production by Pseudomonas fluorescens WH6 Results in Resource Reallocation from Secondary Metabolite Production to Rhizocompetence
Source: Microorganisms. 2021 Mar 31;9(4):717. doi: 10.3390/microorganisms9040717 (PMC8067088; doi:10.3390/microorganisms9040717)
Supplement: Supplementary file 1 [file microorganisms-09-00717-s001.zip › Supplemental/Supplemental2-2.pdf]

A. Increased expression of genes involved in denitrification in *gvg* operon mutants

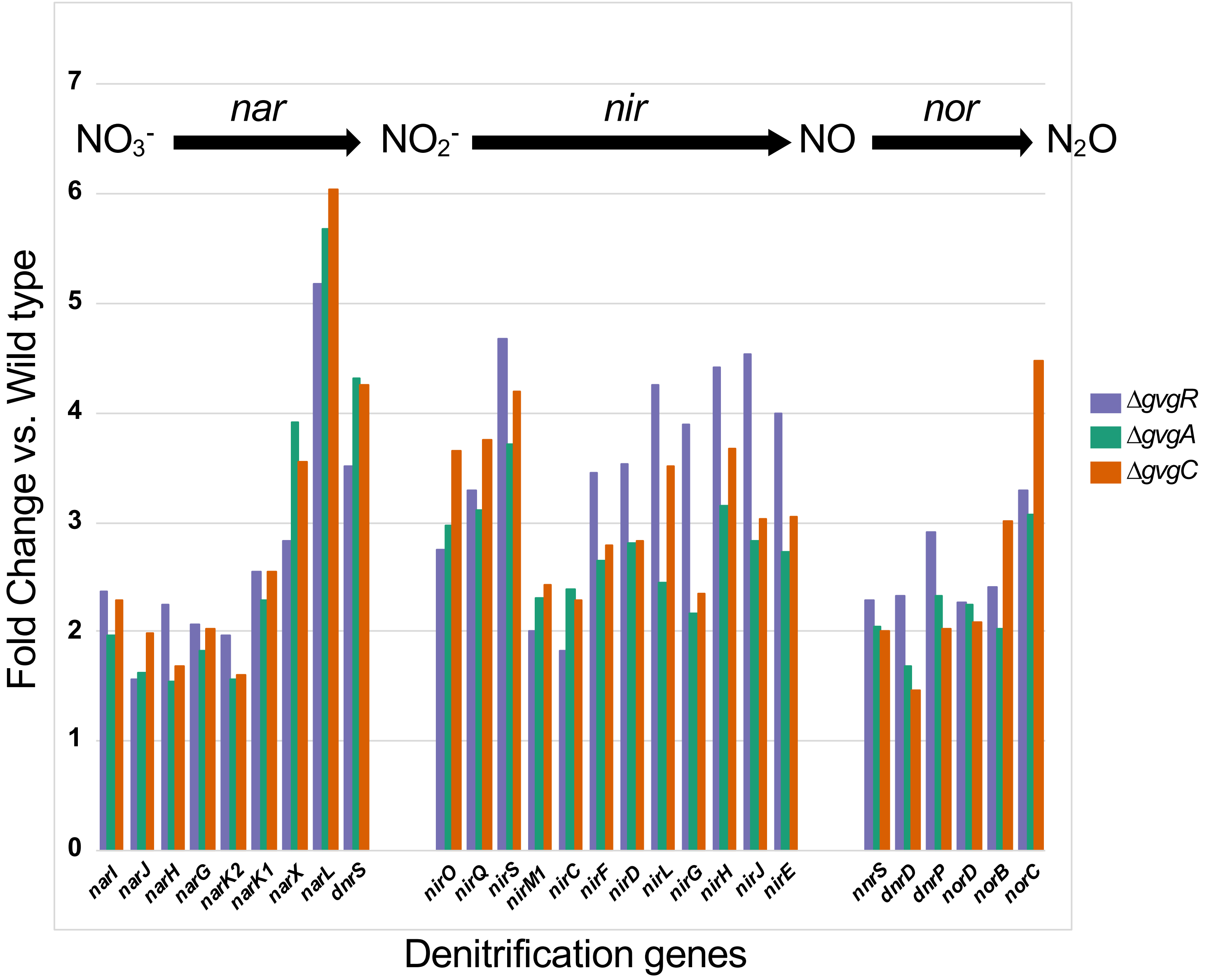

Supplemental Figure 2. Plotting of fold change expression of denitrification genes in Δ*gvgR*, *A*, and *C* strains compared to WT. Schematic above the bar chart displays the steps of denitrification pathway encoded in the WH6 genome.
