# Supplementary material for: Absence of 4-Formylaminooxyvinylglycine Production by Pseudomonas fluorescens WH6 Results in Resource Reallocation from Secondary Metabolite Production to Rhizocompetence
Source: Microorganisms. 2021 Mar 31;9(4):717. doi: 10.3390/microorganisms9040717 (PMC8067088; doi:10.3390/microorganisms9040717)
Supplement: Supplementary file 1 [file microorganisms-09-00717-s001.zip › Supplemental/Supplemental3.pdf]

A. Differentially regulated flagellar genes in the flagellar assembly KEGG pathway

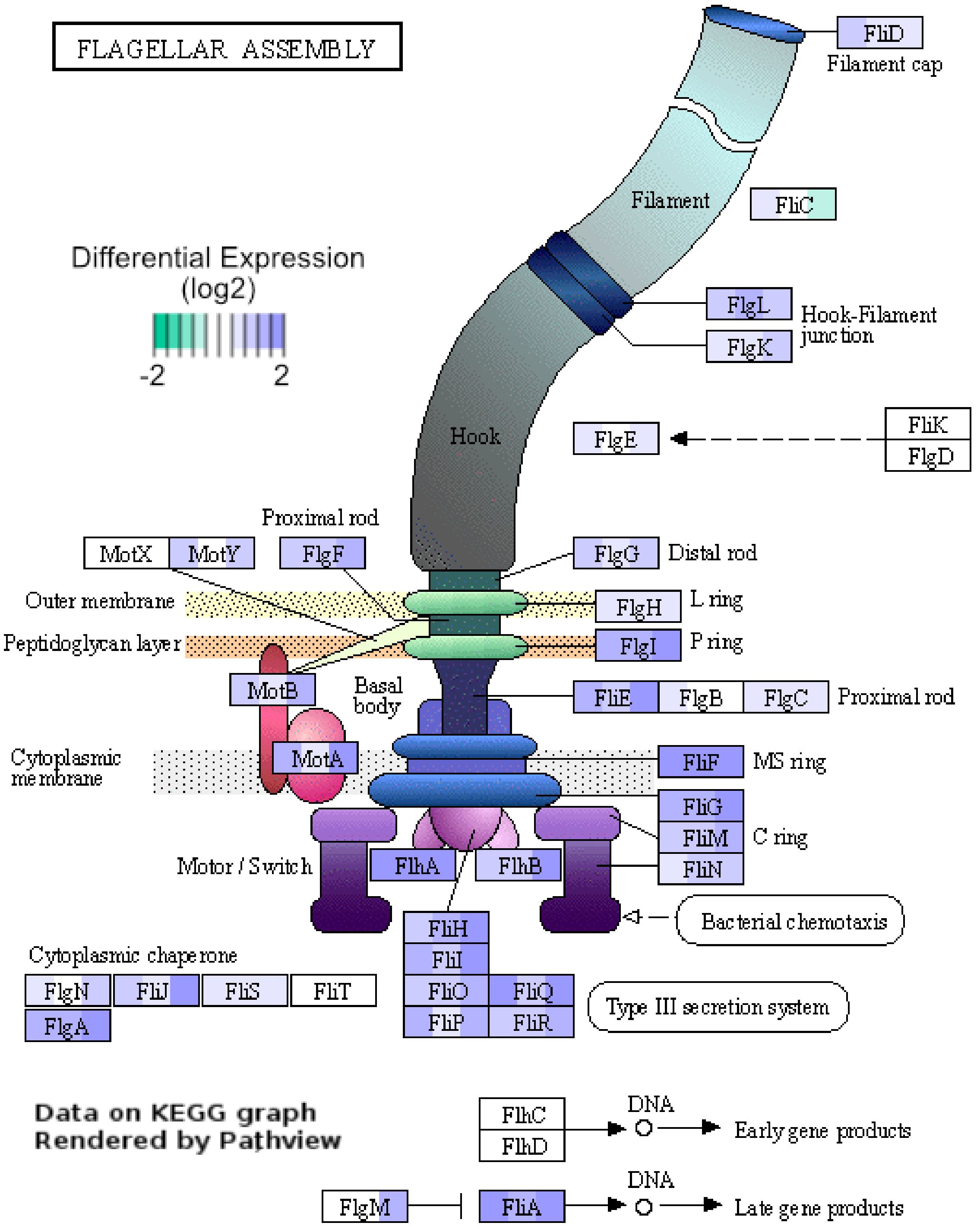

Supplemental Figure 3. Representative example of differentially expressed flagellar assembly genes mapped onto the flagellar assembly KEGG pathway. The strain represented is WH6Δ*gvgA*. Each box represents a gene in the pathway. Within each box there are three colored boxes that represent the fold difference for each of the three replicates.
